# Secure distributed multiple imputation enables missing data inference for private data proprietors

**DOI:** 10.1038/s41746-025-02271-0

**Published:** 2026-01-10

**Authors:** Haris Smajlović, Yi Lian, Qi Long, Ibrahim Numanagić, Xiaoqian Jiang

**Affiliations:** 1 Department of Computer Science, University of Victoria, Victoria, BC Canada; 2 School of Medicine, University of Pennsylvania, Philadelphia, PA USA; 3 University of Texas Health Science Center, Houston, TX USA

**Keywords:** Databases, Software, Statistical methods

## Abstract

Scattered between many healthcare providers across the US, Electronic Health Records (EHR) are extensively used for research purposes. Collaboration and sharing of EHRs between multiple institutions often provide access to more diverse datasets and a chance to conduct comprehensive studies. However, these collaboration efforts are usually hindered by privacy issues that render the pooling of such data at a centralized database impossible. Furthermore, EHRs are often incomplete and require statistical imputation prior to the study. To enable collaborative studies on top of incomplete, private EHRs, here we provide a provably secure solution built with secure multiparty computation (SMC) that provides practical runtimes and accuracy on par with the state-of-the-art, non-secure equivalents. Our solution enables the utilization of distributed datasets as a whole to impute the missing data and conduct collective studies between non-trusting private data proprietors. We demonstrate its effectiveness on various synthetic and real-world datasets, and show that our solution can significantly improve the classification of high-risk patient outcomes during ICU admission.

## Introduction

Electronic Health Records (EHR) have been routinely collected by healthcare providers across the US and extensively used for research purposes. Similarly, claims data from insurance companies are often used in population-based clinical research. Normally, data are stored and managed within the institutions that collect and own them. Storing data locally is generally more feasible logistically, more cost-friendly, and easier for the data-owning entity to access, control, and manage the data. More importantly, local storage helps ensure data sovereignty, and maintain data privacy and security that comply with data protection regulations such as the Health Insurance Portability and Accountability Act (HIPAA)^1,2^. Strong privacy protection helps build confidence in researchers, patients, and other stakeholders to encourage research collaborations in trustworthy medical AI^3^.

Through collaborative research, EHRs and claims data from institutions across diverse geographical locations can form a larger and potentially more representative sample of the US population that could yield more reliable and generalizable research findings^4^. Leveraging distributed data in healthcare research can be particularly beneficial for certain marginalized or minority groups because it allows institutions with very small minority populations to borrow information from others^5–8^. Several large-scale distributed health data networks (DHDN) have been established to facilitate collaborations across multiple institutions. For example, the Sentinel Initiative by the U.S. Food and Drug Administration (FDA) is an effort to monitor the safety of FDA-regulated medical products. The Sentinel can get data from more than a dozen partners including academic medical centers, healthcare systems, and health insurance companies. These data partners collect data in routine operations and maintain control of their own data^9,10^. Another example is the Patient-Centered Scalable National Network for Effectiveness Research (pSCANNER), a national research infrastructure containing data from 13 sites emphasizing comparative effectiveness research^11^. Similarly, data are stored, owned, and governed by each one of the pSCANNER sites without a central data repository.

To enjoy the aforementioned benefits brought by distributed health data, conventional machine learning (ML) methods would require researchers to first “bring data to computation”, transmitting individual patient data from the remote sites to a central data repository and performing centralized ML on aggregated data. However, this is not always permitted for legal reasons or for data privacy and security concerns. In addition, operating data centers that are large enough for centralized storage and computation is financially and logistically challenging, and the consequences are serious if the large data center experiences system failure or data breach^12^. These restrictions and limitations have motivated a broad class of modern distributed ML algorithms that “bring computation to data”^13^. Distributed ML has allowed researchers to take advantage of distributed storage and computational infrastructure and resources, which reduces, if not eliminates, the need for large data centers for EHRs. It also minimizes the need to share sensitive protected health information, complying with legal requirements and improving the privacy and security of healthcare data^14,15^.

Missing data problem is prevalent in real-world EHRs and claims data, therefore DHDNs as well^16,17^. Failure to properly account for missing data will lead to biased inference and prediction results^17–19^. Recent studies further show that missingness in health data tends to harm minority groups disproportionally, exacerbating health inequities and disparities^19^, because the missing information in a minority cohort impacts the accuracy of the downstream studies for that population more severely than for a well-represented one. Also, the discrepancy in representation between different minority groups varies across different factors (e.g., the younger population tends to be generally underrepresented, regardless of their lineage)^19^.

The missing data in EHRs is classified as either *missing completely at random* (MCAR), *missing at random* (MAR), or *missing not at random* (MNAR). The variable is considered missing *at random* if its missingness depends on other variables in the dataset, and missing *not at random* if its missingness is determined by the variable itself. Otherwise, the data is considered MCAR. Complete case analysis that excludes observations with missing values is a valid approach if data is MCAR^18^. However, data that are MAR need to be properly imputed to recover unbiased analysis results^18^.

Multiple imputation (MI) is a popular imputation technique that, in general, works by replacing missing values with predicted values multiple times and combining the analysis results acquired from these imputed datasets. However, since DHDNs comprise data from multiple institutions, the missing data problems could potentially be more complex due to various heterogeneities between the institutions. Compared to the large body of literature on distributed ML algorithms, distributed MI that can handle missing data problems in DHDNs has not received as much attention. In principle, MI algorithms rely on various statistical ML models to impute missing observations. Therefore, distributed MI algorithms should enjoy the same benefits mentioned earlier as model-based distributed analysis. In addition, data sources with either small sample sizes or a small number of observed values due to high missing rates can borrow information from other data sources. To our knowledge, several distributed MI algorithms designed for MAR data have been proposed and have been shown to outperform MI conducted independently at each site^17^. In addition, a distributed MI algorithm for MNAR data that also demonstrates superiority over independent MI algorithms^20^. However, these approaches are not provably secure as they reveal intermediate results that can leak private information. One such example is the Gramian matrix that is revealed by these approaches, which can be used to completely reconstruct private data whenever the number of individuals is less than or equal to the number of training features in some party.

Here, for the first time, we offer a provably secure imputation of the missing data (Secure MICE) in distributed EHR that reveals only the final analysis result. We enabled an otherwise non-secure, centralized *multiple imputation with chained equations* (MICE) algorithm to be executed in secure distributed contexts. Our solution utilizes secure multiparty computation (SMC)^21^ and multiparty homomorphic encryption (MHE)^22^ technologies and provides an accuracy on par with the equivalent non-secure solutions, where the data is pooled into a single cohort. We evaluated our solution on both the MAR and MNAR data, for completeness, and compared them to two non-secure, centralized variants of the MICE algorithm, one with better performance and the other with state-of-the-art accuracy^23^. We used Sequre^24,25^, a framework for high-performance, SMC, to implement our SMC-based algorithms and extended it with MHE protocols to enable the development of our MHE-based solutions. As a result, we obtained practical runtimes of only a few seconds for small-scale solutions and less than 15 s for a large-scale solution. Finally, we showcase a real-life example where our solution enables up to 10% more accurate prediction of death within 48 h of intensive care unit (ICU) admission (Box 1), compared to otherwise non-distributed case where each site computes individually on top of its own data, by imputing and holistically utilizing a large-scale sample of an incomplete Medical Information Mart for Intensive Care (MIMIC) dataset^26^.

In general, we expect Secure MICE to help wholly utilize the private distributed datasets to impute the missing data and enrich the collective statistical studies, thus broadening the data sharing and collaboration efforts between medical institutions and other private data proprietors with incomplete datasets.

## Results

Box 1 The implications of secure distributed imputation *Findings:* The holistic utilization of private distributed datasets, enabled by Secure MICE, improves the prediction power of the final analysis compared to utilizing the private datasets separately (i.e., without data sharing). For example, the MIMIC-IV dataset contains only 305 records of patients who passed away within 48 h of ICU admission that have no missing data in any of the 17 selected variables from demographics, vitals, Glasgow Coma Scale metrics, and laboratory measurements. In other words, only a small fraction of records can be practically utilized for training a supervised learning model to predict patients’ risk of death, and this fraction of data is expected to be even smaller in private medical datasets. Our study shows that secure training on top of distributed data, with secure imputation and data sharing, enables the improved imputation of missing data and results in an average 10% better AUC and accuracy when predicting the risk of death of a recently admitted patient, compared to utilizing only enclosed datasets without data sharing.

*Implications:* Holistic imputation and utilization enabled by Secure MICE would correctly re-classify up to *10 additional high-risk patients per 100 ICU admissions*. This demonstrates the real-world benefit of secure distributed imputation—unlocking predictive power hidden across private and incomplete datasets—without compromising data privacy.

### Experiments setup

We adopted the experiment setup from the previous work^17^, which includes four simulations of data MAR and two real-data studies. While our approach is based on supervised learning and, as such, best suited for imputing the data MAR, we still added two simulation studies on top of data MNAR, for completeness. Each study follows the same pattern. First, the incomplete dataset of a different number of individuals and variables, such as demographic and disease information, is encrypted and pooled together from multiple study participants. The missing data in the pooled dataset is then imputed multiple times to form several independent complete datasets, on top of which different regression models are trained as part of the *final analysis*. The trained models’ weights are then combined via Rubin’s rules to produce a final regression model that is used to assess performance. Each step of the study is done on encrypted data without revealing any meaningful information apart from the final analysis output. Most studies in this work used linear regression as a final analysis model. The only exception is the second real-data study, which used logistic regression for a binary outcome variable. The quality of the final linear regression is measured as a *mean absolute difference* and a *standard deviation of the absolute difference* between the predicted outcome and the ground truth, while the quality of the logistic regression was measured as a combination of *accuracy* and *area-under-curve* (AUC). We also measured the bias $\parallel {\mathbb{E}}{\mathbf{\Theta }}-\mathop{{\mathbf{\Theta }}}\limits^{ \sim }{\parallel }_{2}$, standard deviation $\sqrt{{\mathbb{E}}\parallel {\mathbf{\Theta }}-{\mathbb{E}}{\mathbf{\Theta }}{\parallel }_{2}^{2}}$, and the mean-squared error $\sqrt{{\mathbb{E}}\parallel {\mathbf{\Theta }}-\widetilde{{\mathbf{\Theta }}}{\parallel }_{2}^{2}}$ of the regression weights **Θ** and their ground truth $\widetilde{{\mathbf{\Theta }}}$, where possible. To assess the quality of imputation alone, we additionally measured the mean absolute difference and standard deviation between imputed datasets and their ground truth in the simulation studies with incomplete continuous variables, and accuracy and AUC for the ones with incomplete binary variables. This assessment is not possible in the real-data studies, however, because the ground truth of the missing data is unknown. Finally, we also measured the runtime and network overhead where applicable.

#### Simulation studies

The first simulation (Table 1) is conducted on top of ten variables drawn from a normal distribution ${\mathcal{N}}(0,1)$, and one variable made incomplete uniformly at random with a missingness rate of 30%. The second simulation (Table 2) is the same, with the incomplete variable being a binary variable drawn from a Bernoulli distribution ${\mathcal{B}}(1,0.5)$ instead. The rest of simulation studies (Tables 3–6) have only two variables, *X*_1_ and *X*_2_, with the second variable drawn from a uniform distribution ${\mathcal{U}}(-3,3)$ and the first either from a normal distribution ${\mathcal{N}}(0.2-0.5{X}_{2},1)$ in the third and fifth simulation, or from a Bernoulli distribution ${\mathcal{B}}(1+{e}^{-0.2+0.5{X}_{1}})$ in the fourth and sixth simulation, with the missingness rate from 50% to 60%. Each simulation is benchmarked for a different number of individuals (500 and 5000 for our experiments). The outcome variable (i.e., the ground truth of final regression analysis) in each simulation study is obtained as *Y* = **Θ**_0_ + ∑_*i*_*X*_*i*_**Θ**_*i*_ + *ϵ*, where *X*_*i*_ are the variables; **Θ**_*i*_ the ground truth linear regression weights (set to **1** in our experiments), and *ϵ* is drawn from ${\mathcal{N}}(0,({{\mathbf{\Theta }}}_{0}+{\sum }_{i}{X}_{i}{{\mathbf{\Theta }}}_{i})/100)$. The outcome variable is computed on a complete dataset, before removing the missing data. In each study, five multiple imputations are used (i.e., each dataset is imputed five times and five independent regression models are trained as a part of a final analysis). The simulation and real-data studies are independently benchmarked 100 and 5 times, respectively.

**Table 1:** Scenario 1: Single continuous incomplete variable missing at random, 9 continuous complete variables, 100 random runs, 30% missing rate

		Final analysis						Imputation		Performance	
	Technology	*Θ* bias	*Θ* SD	*Θ* rMSE	$| \widehat{{\rm{y}}}-{\rm{y}}|$ (*μ*)	$| \widehat{{\rm{y}}}-{\rm{y}}|$ (*σ*)	Discr.	$| \widehat{{\rm{y}}}-{\rm{y}}|$ (*μ*)	$| \widehat{{\rm{y}}}-{\rm{y}}|$ (*σ*)	Time (s)	Net (MB)
500 inds.	Python	0.045	10^-4^	0.045	0.041	0.034	0	**0.496**	0.073	**0.061**	N/A
	PyMICE	**0.042**	1.57 × 10^-16^	**0.042**	**0.040**	**0.032**	N/A	0.498	**0.034**	0.223	N/A
	SMC-MICE	0.045	4.18 × 10^-4^	0.045	0.041	0.034	0	**0.496**	0.073	0.090	**4.737**
	MHE-MICE	0.065	3.91 × 10^-4^	0.065	0.044	0.035	0	**0.496**	0.071	1658	19,834
5000 inds.	Python	0.025	4.00 × 10^-16^	0.025	0.031	0.027	0	0.814	0.601	**0.149**	N/A
	PyMICE	**0.019**	4.15 × 10^-16^	**0.019**	**0.027**	**0.025**	N/A	**0.813**	**0.600**	0.205	N/A
	SMC-MICE	**0.019**	1.15 × 10^-4^	**0.019**	0.029	0.026	0	**0.813**	0.601	0.536	44.641
	MHE-MICE	**0.019**	1.13 × 10^-4^	**0.019**	0.029	0.026	0	**0.813**	0.601	2129	32,839

The bolded values denote the best result in each column.

**Table 2:** Scenario 2: Single binary incomplete variable missing at random, 9 continuous complete variables, 100 random runs, 30% missing rate

		Final analysis						Imputation		Performance	
	Technology	Θ bias	Θ SD	Θ rMSE	$| \widehat{{\rm{y}}}-{\rm{y}}|$ (*μ*)	$| \widehat{{\rm{y}}}-{\rm{y}}|$ (*σ*)	Discr.	Accuracy	AUC	Time (s)	Net (MB)
500 inds.	Python	0.268	5.09 × 10^-16^	0.268	0.141	0.092	0	**0.466**	0.477	**0.063**	N/A
	PyMICE	**0.035**	4.00 × 10^-16^	**0.035**	**0.040**	**0.031**	N/A	0.460	0.500	0.225	N/A
	SMC-MICE	0.052	3.12 × 10^-10^	0.052	0.049	0.038	0	**0.466**	**0.432**	0.335	**14.019**
	MHE-MICE	0.053	2.91 × 10^-7^	0.053	0.050	0.038	0	**0.466**	**0.432**	1753	17,061
5000 inds.	Python	0.263	4.71 × 10^-16^	0.263	0.134	0.078	0	0.508	0.509	0.143	N/A
	PyMICE	**0.011**	4.84 × 10^-16^	**0.011**	**0.024**	**0.025**	N/A	**0.510**	0.496	0.212	N/A
	SMC-MICE	0.012	3.30 × 10^-10^	0.012	0.026	0.026	0	0.508	**0.475**	3.052	137.398
	MHE-MICE	0.012	8.31 × 10^-7^	0.012	0.026	0.026	0	0.508	**0.475**	2321	27,038

The bolded values denote the best result in each column.

**Table 3:** Scenario 3: Single continuous incomplete variable missing at random, single continuous complete variable, 100 random runs, 50% missing rate

		Final analysis						Imputation		Performance	
	Technology	Θ bias	Θ SD	Θ rMSE	$| \widehat{{\rm{y}}}-{\rm{y}}|$ (*μ*)	$| \widehat{{\rm{y}}}-{\rm{y}}|$ (*σ*)	Discr.	$| \widehat{{\rm{y}}}-{\rm{y}}|$ (*μ*)	$| \widehat{{\rm{y}}}-{\rm{y}}|$ (*σ*)	Time (s)	Net (MB)
500 inds.	Python	0.014	5.24 × 10^-4^	0.014	**0.028**	0.019	**0**	0.392	0.238	**0.023**	N/A
	PyMICE	0.014	2.22 × 10^-16^	0.014	**0.028**	**0.018**	N/A	0.393	**0.235**	0.046	N/A
	SMC-MICE	**0.013**	2.35 × 10^-3^	**0.013**	**0.028**	**0.018**	**0**	0.392	0.238	0.038	**1.686**
	MHE-MICE	0.016	2.40 × 10^-3^	0.016	0.031	0.021	**0**	**0.388**	0.248	285.9	9,629
5000 inds.	Python	**0.002**	3.09 × 10^-4^	**0.002**	**0.021**	**0.013**	**0**	**0.494**	0.074	0.086	N/A
	PyMICE	0.015	**0.0**	0.015	**0.021**	0.014	N/A	**0.494**	**0.072**	**0.031**	N/A
	SMC-MICE	**0.002**	4.06 × 10^-2^	0.040	0.040	0.019	**0**	**0.494**	0.074	0.206	**16.176**
	MHE-MICE	0.069	2.40 × 10^-2^	0.069	0.051	0.027	**0**	**0.494**	0.087	1910	86,628

The bolded values denote the best result in each column.

**Table 4:** Scenario 4: Single binary incomplete variable missing at random, single continuous complete variable, 100 random runs, 50% missing rate

		Final analysis						Imputation		Performance	
	Technology	Θ bias	Θ SD	Θ rMSE	$| \widehat{{\rm{y}}}-{\rm{y}}|$ (*μ*)	$| \widehat{{\rm{y}}}-{\rm{y}}|$ (*σ*)	Discr.	Accuracy	AUC	Time (s)	Net (MB)
500 inds.	Python	0.378	**0.0**	0.378	0.19	0.026	0	0.636	0.466	**0.024**	N/A
	PyMICE	0.010	2.48 × 10^-16^	0.010	**0.019**	**0.015**	N/A	0.632	0.631	0.047	N/A
	SMC-MICE	0.012	3.85 × 10^-3^	0.012	0.023	**0.015**	0	0.632	**0.719**	0.231	**8.359**
	MHE-MICE	0.028	2.50 × 10^-3^	0.028	0.042	0.026	0	**0.640**	**0.719**	470.4	8298
5000 inds.	Python	0.348	2.48 × 10^-16^	0.348	0.184	0.121	0	**0.529**	0.510	0.081	N/A
	PyMICE	**0.004**	**0.0**	**0.004**	**0.024**	**0.014**	N/A	0.525	0.500	**0.031**	N/A
	SMC-MICE	0.060	1.08 × 10^-1^	0.123	0.105	0.045	0	0.518	**0.467**	2.016	86.63
	MHE-MICE	0.082	4.97 × 10^-3^	0.083	0.087	0.038	**0**	0.523	**0.467**	1737	60,778

The bolded values denote the best result in each column.

**Table 5:** Scenario 5: Single continuous incomplete variable missing not at random, single continuous complete variable, 100 random runs, 55% missing rate

		Final analysis						Imputation		Performance	
	Technology	Θ bias	Θ SD	Θ rMSE	$| \widehat{{\rm{y}}}-{\rm{y}}|$ (*μ*)	$| \widehat{{\rm{y}}}-{\rm{y}}|$ (*σ*)	Discr.	$| \widehat{{\rm{y}}}-{\rm{y}}|$ (*μ*)	$| \widehat{{\rm{y}}}-{\rm{y}}|$ (*σ*)	Time (s)	Net (MB)
500 inds.	Python	**0.002**	1.11 × 10^-16^	**0.002**	0.055	**0.016**	**0**	**0.783**	0.604	**0.013**	N/A
	PyMICE	0.004	1.11 × 10^-16^	0.004	0.055	0.017	N/A	**0.783**	0.604	0.023	N/A
	SMC-MICE	0.003	9.70 × 10^-3^	0.010	**0.052**	0.017	**0**	**0.783**	0.604	0.040	**1.700**
	MHE-MICE	0.013	1.34 × 10^-2^	0.019	0.060	0.023	**0**	**0.783**	0.602	180.3	11,045
5000 inds.	Python	0.011	1.11 × 10^-16^	**0.011**	**0.074**	**0.016**	**0**	0.797	0.618	0.089	N/A
	PyMICE	0.030	**0.0**	0.030	**0.074**	**0.016**	N/A	0.797	**0.617**	**0.030**	N/A
	SMC-MICE	0.022	8.32 × 10^-2^	0.086	0.101	0.045	**0**	0.797	0.618	0.224	**16.359**
	MHE-MICE	**0.010**	6.23 × 10^-2^	0.063	0.095	0.037	**0**	**0.796**	**0.617**	1373	115,759

The bolded values denote the best result in each column.

**Table 6:** Scenario 6: Single binary incomplete variable missing not at random, single continuous complete variable, 100 random runs, 60% missing rate

		Final analysis						Imputation		Performance	
	Technology	Θ bias	Θ SD	Θ rMSE	$| \widehat{{\rm{y}}}-{\rm{y}}|$ (*μ*)	$| \widehat{{\rm{y}}}-{\rm{y}}|$ (*σ*)	Discr.	Accuracy	AUC	Time (s)	Net (MB)
500 inds.	Python	0.308	1.57 × 10^-16^	0.308	0.127	0.105	0	0.498	0.497	**0.021**	N/A
	PyMICE	**0.008**	**0.0**	**0.008**	0.023	0.022	N/A	**0.692**	0.685	0.027	N/A
	SMC-MICE	0.036	3.03 × 10^-3^	0.036	0.056	0.043	0	0.639	**0.738**	0.307	**7.510**
	MHE-MICE	**0.008**	1.92 × 10^-3^	**0.008**	**0.022**	**0.021**	0	0.639	0.737	285.0	9,756
5000 inds.	Python	0.064	**0.0**	0.064	0.034	0.017	0	0.525	**0.527**	0.273	N/A
	PyMICE	**0.010**	2.22 × 10^-16^	**0.010**	**0.016**	**0.012**	N/A	**0.557**	0.500	**0.036**	N/A
	SMC-MICE	0.131	1.86 × 10^-2^	0.132	0.030	0.021	0	**0.557**	0.479	2.272	77.68
	MHE-MICE	0.144	2.19 × 10^-3^	0.145	0.033	0.023	**0**	0.556	0.478	1246	70,265

The bolded values denote the best result in each column.

#### Real-data studies

We used Secure MICE to predict the *arrival-to-computed tomography time* and *death within 48* *h* using two large patients’ cohorts (Tables 7 and 8). For the first real-data study, the data from the Georgia Coverdell Acute Stroke Registry (GCASR) is used with 15 out of 203 selected variables (five continuous and ten binary based on previous work^17^) and 68,287 patients. Each continuous variable and seven binary variables are incomplete, and the missingness rate ranges between 0.035% and 53.84%. For the second real-data study, we used a sample of 94,459 patients from the MIMIC dataset^26^, for which we were able to curate 17 out of 164 continuous variables, such as basic demographics like age, ICU type, ICU admission time, and length of stay; vitals like heart rate and systolic/diastolic blood pressure, mean arterial pressure, respiratory rate, body temperature, and oxygen saturation; four Glasgow Coma Scale metrics for neurological assessment (total score and eye/verbal/motor responses); and laboratory measurements like glucose and blood pH, with a missing rate of 57% on average (ranging from none to 84% per variable). This sample included 3445 patients who died within 48 h of ICU admission and to reduce the prediction bias, we sampled the same number of patients who survived to come up with the total number of 6890 patients in the training dataset.

**Table 7:** Real-data scenario (GCASR): 90,000 individuals, 5 continuous and 10 binary incomplete (missing) variables with a missing rate ranging from 0.035% to 53.84%

	Final analysis			Performance	
Technology	$| \widehat{{\rm{y}}}-{\rm{y}}|$ (*μ*)	$| \widehat{{\rm{y}}}-{\rm{y}}|$ (*σ*)	Discr.	Time (s)	Net (MB)
Python	0.306	0.333	1	23.185	N/A
PyMICE	**0.305**	**0.332**	N/A	98.716	N/A
SMC-MICE	**0.305**	**0.332**	**0**	**12.108**	**712**
MHE-MICE	**0.305**	**0.332**	**0**	2017	19,264

The bolded values denote the best result in each column.

**Table 8:** Real-data scenario (MIMIC) for prediction of death within 48 h after ICU admission using baseline demographics, neurological assessments, vitals, and laboratory measurements within the first 2 h of admission: 6890 individuals, 17 continuous variables with a missing rate of up to 84%

	Final analysis			Performance	
Technology	AUC	Accuracy	Discr.	Time (s)	Net (MB)
Python	0.888	**0.786**	**6**	**1.701**	N/A
PyMICE	**0.892**	0.783	N/A	3.907	N/A
SMC-MICE	0.886	0.780	**5**	285.450	**2647**
MHE-MICE	0.886	0.780	**5**	6440	88,920

The bolded values denote the best result in each column.

### Benchmarked solutions and implementation details

We implemented two secure solutions for MICE, one based on SMC and the other on MHE. Additionally, we implemented two non-secure solutions to compare against. The first one is a raw Python implementation of MICE using off-the-shelf linear and logistic regression for imputation and final analysis, and the second one is an off-the-shelf MICE algorithm from Python’s scikit-learn library^23^. There is no clear winner between the two non-secure solutions, but the latter is generally expected to have better accuracy, while the former has better performance. The Python-based solutions are tested in an offline, non-secure context on top of plain, non-encrypted data, while the secure solutions are tested in a secure distributed setup, on top of encrypted data, with two computing parties aided by a *trusted dealer*.

We implemented both secure solutions in Sequre^24,25^ in less than 550 lines of Pythonic code. Sequre’s compile-time optimizations for network overhead reduction, as well as the low-level performance optimizations such as modulo operator customization and exposing data-level parallelism, are mainly responsible for achieving the practical runtimes. Also, Sequre’s configurable fixed-point arithmetic allowed us to reduce the truncation error noise in SMC. In particular, we used 192-bit long integers, with 32 bits reserved for the fractional part, 64 bits for a whole fixed-point value, and 64 bits of padding for statistical security. To obtain similar accuracy in MHE, we adhered to common CKKS parameters with 128-bit security, enabling 8192 slots with a default scale of 2^34^, which provides a good balance between performance and accuracy^27,28^. Lastly, all experiments were done on a single 12-core Intel Core i7-8700 CPU at 3.20GHz and 64 GB of RAM. To simulate a multiparty setup, the UNIX sockets were used to connect multiple processes—each process corresponding to a separate computing party. Nevertheless, Sequre allows easy deployment across arbitrary network architectures and, as such, will facilitate seamless integration of our solution across multiple institutions.

### Evaluation

The imputation and the final study quality of secure solutions are on par or slightly better than the offline solutions in all simulation studies. We note that our goal was not to improve the existing MICE algorithms but to design their secure equivalents with on-par accuracy and performance for the first time. The imputation accuracy is slightly worse (<0.006) only in studies where a categorical variable is imputed (Table 2 and Table 4) due to approximation algorithms (Chebyshev approximation) employed in secure variants of logistic regression. Similarly, the quality of the final study is only fractionally worse (0.001–0.063) in secure solutions—the offset that can be further attributed to approximation errors that are unavoidable in the security schemes that we employ^24,29^.

We simulated the distributed environment for the last real-data study (the prediction of death within 48 h on top of the MIMIC dataset) by splitting the data between three multiple sites. We first conducted a separate, independent run at each site, without imputation and data sharing. Each site’s data consisted of about 100 patients, since only 305 patients in the MIMIC dataset had complete data (i.e., no missing variables). As such, the accuracy and AUC of predicting the risk of death of recently admitted patients were 0.70 and 0.80, respectively. Then we conducted the same study using our secure solutions where the data of all 6890 patients was imputed and utilized for training and ultimately achieved an accuracy and AUC of 0.77 and 0.88, respectively (Table 8). In other words, our solution improves the classification of 10% additional high-risk patients per a number of ICU admissions. Moreover, apart from the MHE variant, which is slower for this amount of data due to under-utilization of its packing mechanism in which operations are executed over encrypted arrays in a SIMD-like manner^29^, the runtimes of SMC solutions are generally small (12 s for GCASR and 285 s for the MIMIC dataset). This is an important practical result since secure solutions are generally known to incur large performance overhead^24^. The reason for the slowdown in MIMIC-based experiment, even though it runs a smaller dataset than that of GCASR, is that the final outcome variable is binary and, thus, the final analysis model—utilized on a large, imputed dataset—uses logistic regression that employs the expensive polynomial approximations for the logistic sigmoid. Nevertheless, computing the risk score for a single patient (i.e., single inference) requires only 15 μs in SMC and 1 ms in MHE variants.

### Discrepancy analysis

To further assess the quality of our imputation algorithms, we measured a *number of discrepancies*^17^ with respect to an off-shelf MICE algorithm from scikit-learn library as a base algorithm. In short, a variable in the final linear regression study has a discrepancy between the two MICE algorithms (target and base algorithm) if and only if its statistical significance is less or equal to 0.05 in the base algorithm and either its statistical significance in the target algorithm is larger than 0.05 or its weights in the two algorithms have the opposite signs. The smaller number of discrepancies is desired since it indicates similar imputation quality between the two algorithms. In our measurements, we observed one discrepancy in the offline Python implementation of MICE in the GCASR study, compared to no discrepancies in the secure counterpart. We also measured six discrepancies in an offline Python study on top of the MIMIC dataset, while our secure equivalent produced five. We measured no discrepancies in any other solution across all studies. Counting the number of discrepancies is particularly useful when there is no ground truth to measure the quality of imputation, such as in real-data studies.

## Discussion

We enable provably secure statistical studies on top of private, incomplete distributed datasets while maintaining data privacy. Specifically, we used SMC and MHE to implement a secure distributed variant of *multiple imputation with chain equations (MICE)* procedure and enable imputing the missing data in a distributed setup and performing statistical analysis on top of it without revealing any meaningful information apart the final outcome to the study participants. Our solution proved to have practical performance and an on-par accuracy with the standard, non-secure, and centralized implementations of MICE, where data is assumed to be pooled together in a single cohort and which is often hindered in practice due to privacy concerns. For example, predicting the risk of death of a recently admitted ICU patient in the MIMIC dataset^26^, distributed across multiple sites that cannot directly share their data due to privacy, is 10% more accurate with our solution because it enables the distributed dataset to be utilized securely as a pooled cohort, in contrast to each site doing the prediction on top of their own dataset, without data sharing. Moreover, the high-level expressiveness of our solution allows for an easy adoption and deployment of our protocols, even when the size and resources of the institute employing them are limited. This is because Sequre—the secure programming framework we utilized—is written in a high-level, Pythonic syntax, oblivious of SMC or MHE-specific concerns, and is automatically optimized for performance, which allowed our experiments to be done on standard office hardware.

Our solution follows an honest-but-curious trust model, where study participants are expected to faithfully follow the execution protocol without altering either the algorithm or the data, but are allowed to arbitrarily interact with the data they possess or receive throughout the computation. Also, as the foundational MICE algorithm is apt for imputing the MAR data only, our secure methods are limited to the same missingness type, too. To increase their versatility, provide different security guarantees, and potentially achieve even better runtime and accuracy, we plan to extend our solution with more accurate secure algorithms for imputing the MNAR data, and support for malicious-safe protocols and trusted executing environments such as Intel’s SGX^30^. Also, as our solution currently supports only regression models in the final analysis, we plan to add support for other machine learning models and, in particular, deep learning-based models.

## Methods

### Missing data imputation

**Fig. 1:**
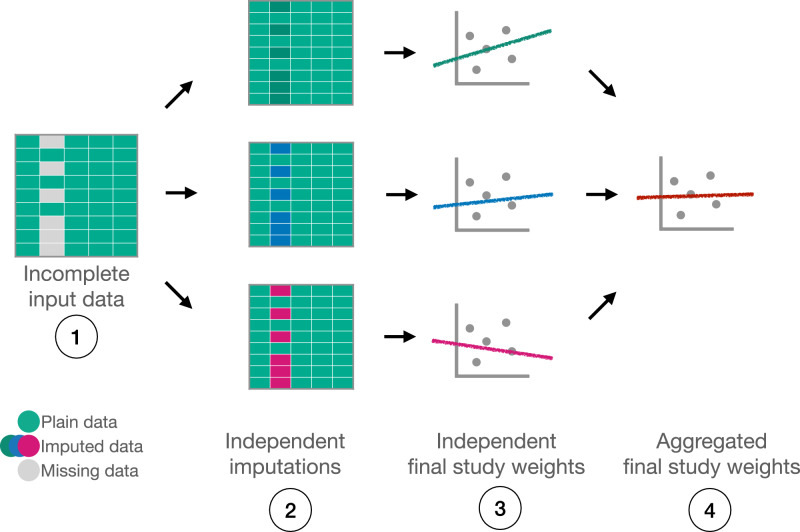
Multiple imputation. The missing data is independently imputed multiple times to address the uncertainty of imputation. Then, a set of independent studies is done on top of imputed datasets, and their parameters are combined using Rubin’s rules to produce a final study. For example, if the final study involves doing a linear regression on top of a dataset, then multiple linear regression models will be independently trained and their coefficients combined, usually through some aggregation, into a final linear regression model.

*Multiple imputation* (MI) addresses the uncertainty of the single imputation by probabilistically imputing data multiple times before conducting a study. The study is then done independently over each imputed dataset, and the results are combined via Rubin’s rules, usually in the form of an aggregate statistic of the underlying studies’ coefficients (Fig. 1)^31^. Whenever more than one variable in the initial dataset is incomplete during a single imputation, data is imputed iteratively, one variable at a time, while re-using the complete data from the previously imputed variables. This procedure is called *multiple imputation with chained equations* (MICE).

### Privacy enhancing technologies

*Privacy-enhancing technologies* protect data privacy throughout the computation. Prominent examples include *differential privacy* (DP)^32^, SMC^21^, *homomorphic encryption* (HE)^33^, and MHE^22^. Here, we focus on SMC and MHE, the technologies that enable computation on top of distributed data privately held by multiple stakeholders without disclosing any meaningful information to each other. Specifically, SMC enables computation on top of private distributed datasets by *secret sharing*^34^ and pooling all private data partitions into a single encrypted tensor (usually in the form of a matrix) and employing a set of specialized routines that enable computation on top of such shared data. As SMC comes in many variants, we settle for *additive secret-sharing* with honest-but-curious stakeholders (meaning that the parties will execute the provided code correctly but might try to infer information about the other parties’ data), aided by a trusted dealer^34^.

MHE, on the other hand, combines SMC with homomorphic encryption—another fundamental cryptographic primitive. Homomorphic encryption (HE) is a form of encryption that allows direct computations over encrypted data without decryption. In this work, we rely on the Cheon-Kim-Kim-Song (CKKS) scheme^29^, which sacrifices *perfect correctness* for improved performance and which encodes vectors of continuous values. This scheme supports vector additions, multiplications, and rotations, and any operation is performed simultaneously on all the vector values akin to the “single instruction, multiple data” (SIMD) instructions. To maintain the ciphertext size and scale (values are scaled by a constant before encryption to ensure a high level of precision), ciphertexts have to be *rescaled* after any multiplication and *relinearized* after multiplication with another ciphertext. After a certain number of multiplications, the ciphertext needs to be *refreshed* through a *bootstrapping* procedure to ensure correct decryption. While this operation is prohibitively expensive in the standard CKKS scheme, in MHE, it can be substituted with an interactive protocol where ciphertexts are transformed into secret shares and re-encrypted. Using a similar approach, a ciphertext can be converted into additive shares^35^, which can be used for SMC operations. While HE enables efficient polynomial operations on large-scale vector operations, non-polynomial operations such as comparisons, square root, and division can be efficiently evaluated in the secret-sharing variant of SMC.

### Secure MICE algorithm

**Fig. 2:**
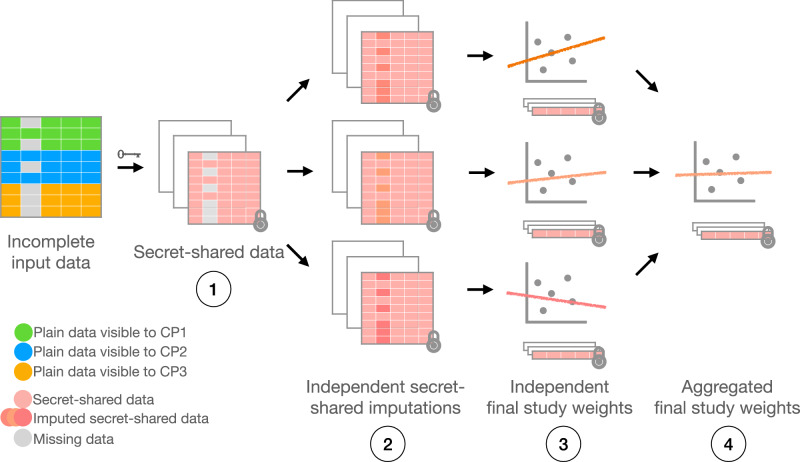
Multiple imputation via SMC. The input data is first secret-shared and then imputed and analyzed in SMC context. Each independent study produces secret-shared coefficients that are averaged together without decryption. The result is a final, secure linear regression model that allows inference on top of encrypted data without revealing any meaningful information.

**Fig. 3:**
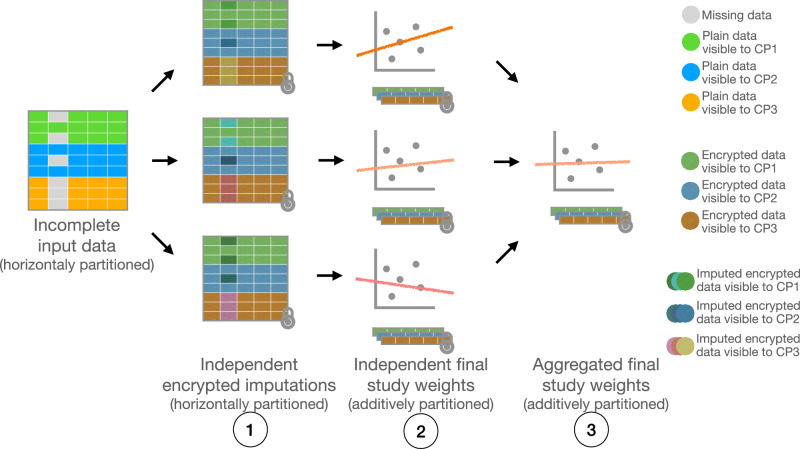
Multiple imputation via MHE. The input data is distributed between the parties and kept in a non-encrypted form, only to be encrypted when needed during the imputation and final analysis. The procedure also benefits from independent, parallel computation on top of local data partitions. This scheme, however, is suitable only for large-scale datasets due to the performance overhead incurred by the underlying, expensive cryptographic scheme that is inherently scalable with respect to data size and the number of computing parties.

We consider a typical distributed use case where the incomplete training data is horizontally divided between the parties (i.e., each party contributes with a different number of individuals and the same number of training features). We note, however, that our solution is also applicable to other distribution types, such as vertical or even additive, where the sum of private data partitions forms the complete dataset. We enabled two variants of secure distributed MICE, one implemented using secure multiparty computation (SMC-MICE; Algorithm 1; Fig. 2) and the other using multiparty homomorphic encryption (MHE-MICE; Algorithm 5; Fig. 3). The former is suitable for small data scales (approximately less than 300,000 individuals) and a number of computing parties, while the latter scales better with the increase of data size or number of parties. Both schemes enable computation on top of encrypted, distributed data without revealing any meaningful information to the study participants or data owners.

Our solution, in both schemes, works conceptually as follows. The training data is first encrypted and pooled together from multiple data owners before being imputed multiple times using linear regression with error (drawn from ${\mathcal{N}}(0,0.01)$) for imputing continuous variables or logistic regression for categorical variables. On top of each imputed dataset, an independent linear or logistic regression is trained as a part of a final study, and the arithmetic average of the resulting models’ coefficients is used as Rubin’s rules to produce the final regression model. We utilized a mini-batched gradient descent with a pre-defined step size and a number of epochs for both linear and logistic regression against the mean-squared and categorical cross-entropy loss, respectively. Additionally, we used a closed-form solution if the number of features is relatively small (less than 4 in our implementations) in linear regression. Each step, in both SMC and MHE variants of MICE, is done on top of the encrypted data without revealing any meaningful information to the parties.

In SMC-MICE, the computing parties first secret share the incomplete training data ([**X**]) and the training labels ([**y**]). Additionally, each party provides the *missigness* mask for its data partition (i.e., the zero-one matrix where 0 denotes missing entry). The missingness masks are pooled into a single matrix (**M**) that remains public throughout execution. The incomplete training data is then imputed multiple times using an SMC implementation of the two aforementioned regression models (i.e., linear for continuous and logistic for categorical variables). Each imputed, secret-shared dataset is used to train an independent final analysis model, which in our case again, is an SMC variant of linear or logistic regression. The secret shares of the models’ weights are then pooled and averaged together to form the final regression model. The details of the SMC-MICE algorithm are provided in Algorithm 1 and Algorithm 2. The former provides a general overview, while the latter gives an insight into a single imputation procedure where, for each incomplete variable, a different SMC regression model is used to infer the missing data. The SMC implementation of linear regression uses Beaver triplets^36^ to enable secure multiplication and computes the secret shares of weights in an otherwise classical manner (see Algorithm 3 for details), using only simple operations such as addition and subtraction (together with multiplication) that are efficient in our security scheme. Also, each additive operation is computed independently at each party without network overhead. For logistic regression, we implemented SMC variants of Chebyshev interpolation to support sigmoid and logarithms that are otherwise hard to compute in SMC. Lastly, we also employed the existing optimization techniques for caching the Beaver triplets^24^ to reduce network consumption.

#### Algorithm 1

Regression analysis via multiple imputation using SMC

INPUT:

$[{\bf{X}}]\in {{\mathbb{Z}}}_{p}^{m\times n}$: secret shared incomplete training data

$[{\bf{y}}]\in {{\mathbb{Z}}}_{p}^{m}$: secret shared training labels

**M** ∈ {0, 1}^*m*×*n*^: public missing data mask

${{\mathcal{M}}}_{im}$: SMC imputation model

${{\mathcal{M}}}_{f}$: SMC final analysis model

*k*: public number of multiple imputations

OUTPUT:

$[{\bf{c}}]\in {{\mathbb{Z}}}_{p}^{n+1}$: secret shared final analysis model coefficients

1: **procedure** SMC_MICE_ANALYSIS $$([{\bf{X}}],[{\bf{y}}],{\bf{M}},{{\mathcal{M}}}_{im},{{\mathcal{M}}}_{f},k)$$

2: $[{\bf{C}}]\leftarrow [{\bf{0}}]\in {{\mathbb{Z}}}_{p}^{k\times (n+1)}$ ⊳Secret shared zeros

3: **for** $$j=\overline{0,\ldots {\mathtt{k}}}$$ **do**

4: $${\mathtt{smc}}\_{\mathtt{mice}}\_{\mathtt{impute}}({{\mathcal{M}}}_{im},[{\bf{X}}],{\bf{M}})$$

5: $${\mathtt{smc}}\_{\mathtt{fit}}({{\mathcal{M}}}_{f},[{\bf{X}}],[{\bf{y}}])$$

6: $${[{\bf{C}}]}_{j}\leftarrow {\mathtt{get}}\_{\mathtt{coeffs}}({{\mathcal{M}}}_{f})$$

7: **end** **for**

8: [**c**] ← smc_rubin([**C**])

9: **return** [**c**]

10: **end** **procedure**

#### Algorithm 2

Imputation algorithm via chained equations via SMC

INPUT:

$${{\mathcal{M}}}_{im}$$: imputation model

$[{\bf{D}}]\in {{\mathbb{Z}}}_{p}^{m\times n}$: secret shared incomplete training data

**M** ∈ {0, 1}^*m*×*n*^: missing data mask

1: **procedure** SMC_MICE_IMPUTE$$({{\mathcal{M}}}_{im},[{\bf{D}}],{\bf{M}})$$

2: *n* ← len([**D**]^⊤^)

3: **for** $$j=\overline{0,\ldots n}$$ **do**

4: **C** ← [**D**]_**M**=1_ ⊳Filter only complete data

5: [**X**] ← [**C**]_:,*k*≠*j*_

6: [**y**] ← [**C**]_:,*j*_

7: $${\mathtt{smc}}\_{\mathtt{fit}}({{\mathcal{M}}}_{im},[{\bf{X}}],[{\bf{y}}])$$

8: $\epsilon \leftarrow {\mathcal{N}}(0,0.01)$ ⊳Draw error from normal distribution

9: $$[\widehat{{\bf{y}}}]\leftarrow {\mathtt{smc}}\_{\mathtt{predict}}({{\mathcal{M}}}_{im},{([{\bf{D}}]\cdot {\bf{M}})}_{:,k\ne j},\epsilon )$$

10: $${[{\bf{D}}]}_{:,j}\leftarrow [\widehat{{\bf{y}}}]$$

11: $${{\bf{M}}}_{:,j}\leftarrow {\bf{1}}\in {{\mathbb{R}}}^{m\times 1}$$

12: **end** **for**

13: **end** **procedure**

#### Algorithm 3

Linear regression via SMC (using batched instead of mini-batched gradient descent to simplify)

INPUT:

$[{\bf{X}}]\in {{\mathbb{Z}}}_{p}^{m\times n}$: secret shared training data

$[{\bf{y}}]\in {{\mathbb{Z}}}_{p}^{m\times 1}$: secret shared training labels

${\mathcal{M}}$: linear regression model that stores initial, secret shared weights $([{{\bf{w}}}_{{\mathcal{M}}}]\in {{\mathbb{Z}}}_{p}^{(n+1)\times 1})$, number of training epochs $({e}_{{\mathcal{M}}}\in {\mathbb{N}})$, and step size $({\eta }_{{\mathcal{M}}}\in {\mathbb{R}})$

1: **procedure** SMC_FIT$$({\mathcal{M}},[{\bf{X}}],[{\bf{y}}])$$

2: $[\widetilde{{\bf{X}}}]\leftarrow ({\bf{X}}\parallel {\bf{1}})$ ⊳Append bias column

3: $$[{\bf{C}}]\leftarrow {[\widetilde{{\bf{X}}}]}^{\top }\times [\widetilde{{\bf{X}}}]$$

4: $$[{\bf{R}}]\leftarrow {[\widetilde{{\bf{X}}}]}^{\top }\times [{\bf{y}}]$$

5: **if** ${\mathtt{len}}({[\widetilde{{\bf{X}}}]}^{\top }) < 4$ **then** ⊳Closed-form solution

6: $$[{{\bf{w}}}_{{\mathcal{M}}}]\leftarrow {[{\bf{C}}]}^{-1}\times [{\bf{R}}]$$

7: **else** ⊳Batched gradient descent

8: **for** $$j=\overline{0,\ldots {e}_{{\mathcal{M}}}}$$ **do**

9: $$[{{\bf{w}}}_{{\mathcal{M}}}]\leftarrow [{{\bf{w}}}_{{\mathcal{M}}}]+([{\bf{R}}]-[{\bf{C}}]\times [{{\bf{w}}}_{{\mathcal{M}}}])\cdot {\eta }_{{\mathcal{M}}}$$

10: **end** **for**

11: **end** **if**

12: **end** **procedure**

#### Algorithm 4

Logistic regression via SMC (using batched instead of mini-batched gradient descent to simplify)

INPUT:

$[{\bf{X}}]\in {{\mathbb{Z}}}_{p}^{m\times n}$: secret shared training data

$[{\bf{y}}]\in {{\mathbb{Z}}}_{p}^{m\times 1}$: secret shared training labels

${\mathcal{M}}$: logistic regression model that stores initial weights $({{\bf{w}}}_{{\mathcal{M}}}\in {{\mathbb{Z}}}_{p}^{(n+1)\times 1})$, number of training epochs $({e}_{{\mathcal{M}}}\in {\mathbb{N}})$, and step size $({\eta }_{{\mathcal{M}}}\in {\mathbb{R}})$

1: **procedure** SMC_FIT $$({\mathcal{M}},[{\bf{X}}],[{\bf{y}}])$$

2: $[\widetilde{{\bf{X}}}]\leftarrow ({\bf{X}}\parallel {\bf{1}})$ ⊳Append bias column

3: **for** $$j=\overline{0,\ldots {e}_{{\mathcal{M}}}}$$ **do**

4: $$[{\bf{A}}]\leftarrow {\sigma }_{cheby}([\widetilde{{\bf{X}}}]\times [{{\bf{w}}}_{{\mathcal{M}}}],(0,1))$$

5: $$[{{\bf{w}}}_{{\mathcal{M}}}]\leftarrow [{{\bf{w}}}_{{\mathcal{M}}}]-{[\widetilde{{\bf{X}}}]}^{\top }\times ([{\bf{A}}]-[{\bf{y}}])\cdot {\eta }_{{\mathcal{M}}}$$

6: **end** **for**

7: **end** **procedure**

The MHE-MICE is conceptually the same as its SMC counterpart (see Algorithm 5 and Algorithm 6). The main difference is in the input data format and the implementation of elementary matrix algebra operations. Namely, the input data to MHE-MICE is initially kept local, non-encrypted at each party, and only encrypted and shared when needed throughout the computation. This, for example, enables the pre-processing steps in the imputation algorithm (Algorithm 6) to be done independently at each party on top of local, non-encrypted data. Generally, any element-wise operation, such as addition, subtraction, and multiplication, is computed in the same manner—independently at each party—as long as the partition sizes of the operands are aligned between the parties. Moreover, computing the invariants in linear regression (i.e., the Gramian matrix ${\widetilde{X}}^{\top }\times \widetilde{X}$ and ${\widetilde{X}}^{\top }\times y$) is also done independently at each party since the product of vertically and horizontally partitioned matrices is an additively partitioned matrix with each partition being a product of corresponding non-encrypted local shares. Some operations, however, require one of the operands to be *aggregated* (i.e., encrypted and shared among the parties) beforehand. For example, matrix multiplication of two additively partitioned matrices requires at least one operand to be aggregated beforehand. The result is then obtained by multiplying each additive share with the aggregated counterpart independently at each party. The aggregation strategy (i.e., deciding whether to aggregate the first or the second operand) directly impacts the partitioning of the result and the performance of all downstream operations. For example, aggregating the weights $[{{\bf{w}}}_{{\mathcal{M}}}]$ instead of training data $\widetilde{X}$ in logistic regression in Algorithm 8 would result in a completely different algorithm downstream. In our particular implementation, it is better to aggregate $\widetilde{X}$ first to avoid aggregating $[{{\bf{w}}}_{{\mathcal{M}}}]$ multiple times within the loop body. Moreover, multiplying vertically partitioned against the horizontally partitioned matrix, as well as two additively partitioned matrices, are the only two matrix multiplication instances encountered in our implementation of linear and logistic regression.

#### Algorithm 5

Regression analysis via multiple imputation using MHE INPUT:

$X\in {{\mathbb{R}}}^{{m}_{i}\times n}$: incomplete training data partition held locally at *i* th party

$y\in {{\mathbb{R}}}^{{m}_{i}}$: training labels partition held locally at *i* th party

$M\in {\{0,1\}}^{{m}_{i}\times n}$: missing data mask held locally at *i*th party

${{\mathcal{M}}}_{im}$: MHE imputation model

${{\mathcal{M}}}_{f}$: MHE final analysis model

*k*: public number of multiple imputations

*N*: number of CKKS slots

${\mathcal{C}}$: CKKS ciphertexts space (i.e., ${({{\mathbb{Z}}}_{p}[X]/(X+1))}^{2}$)

OUTPUT:

${\bf{c}}\in {{\mathcal{C}}}^{\lceil n/N\rceil }$: aggregated final analysis model coefficients

1: **procedure** MHE_MICE_ANALYSIS $$(X,y,M,{{\mathcal{M}}}_{im},{{\mathcal{M}}}_{f},k)$$

2: ${\bf{C}}\leftarrow {\bf{0}}\in {{\mathcal{C}}}^{k\times \lceil (n+1)/N\rceil }$ ⊳CKKS encrypted zeros

3: **for** $$j=\overline{0,\ldots {\mathtt{k}}}$$ **do**

4: $${\mathtt{mhe}}\_{\mathtt{mice}}\_{\mathtt{impute}}({{\mathcal{M}}}_{im},X,M)$$

5: $${\mathtt{mhe}}\_{\mathtt{fit}}({{\mathcal{M}}}_{f},X,y)$$

6: $${{\bf{C}}}_{j}\leftarrow {\mathtt{get}}\_{\mathtt{coeffs}}({{\mathcal{M}}}_{f})$$

7: **end** **for**

8: **c** ← mhe_rubin(**C**)

9: **returnc**

10: **end** **procedure**

#### Algorithm 6

Imputation algorithm via chained equations using MHE

INPUT:

$${{\mathcal{M}}}_{im}$$: imputation model

$D\in {{\mathbb{R}}}^{{m}_{i}\times n}$: incomplete training data partition held locally at *i*th party

$M\in {\{0,1\}}^{{m}_{i}\times n}$: missing data mask held locally at *i*th party

${\mathcal{C}}$: CKKS ciphertexts space (i.e., ${({{\mathbb{Z}}}_{p}[X]/(X+1))}^{2}$)

1: **procedure** MHE_MICE_IMPUTE$$({{\mathcal{M}}}_{im},D,M)$$

2: *n* ← len(*D*^⊤^)

3: **for** $$j=\overline{0,\ldots n}$$ **do**

4: *C* ← *D*_*M*=1_ ⊳Filter only complete data at each party

5: *X* ← *C*_:,*k*≠*j*_

6: *y* ← *C*_:,*j*_

7: $${\mathtt{mhe}}\_{\mathtt{fit}}({{\mathcal{M}}}_{im},X,y)$$

8: $\epsilon \leftarrow {\mathcal{N}}(0,0.01)$ ⊳Draw error from normal distribution

9: $\widehat{y}\leftarrow {\mathtt{mhe}}\_{\mathtt{predict}}({{\mathcal{M}}}_{im},{(D\cdot M)}_{:,k\ne j},\epsilon )$ ⊳Local partition of imputed column

10: $${D}_{:,j}\leftarrow \widehat{y}$$

11: $${M}_{:,j}\leftarrow {\bf{1}}\in {{\mathbb{R}}}^{{m}_{i}\times 1}$$

12: **end** **for**

13: **end** **procedure**

#### Algorithm 7

Linear regression via MHE (using batched instead of mini-batched gradient descent to simplify)

INPUT:

*N*: number of CKKS slots

${\mathcal{C}}$: CKKS ciphertexts space (i.e., ${({{\mathbb{Z}}}_{p}[X]/(X+1))}^{2}$)

$X\in {{\mathbb{R}}}^{{m}_{i}\times n}$: training data partition held locally at *i*th party

$y\in {{\mathbb{R}}}^{{m}_{i}\times 1}$: training labels partition held locally at *i*th party

${\mathcal{M}}$: linear regression model that stores initial, aggregated weights $\left(\left[{{\bf{w}}}_{{\mathcal{M}}}\right]\right.\in {{\mathcal{C}}}^{\lceil (n+1)/N\rceil \times 1}$, number of training epochs $({e}_{{\mathcal{M}}}\in {\mathbb{N}})$, and step size $({\eta }_{{\mathcal{M}}}\in {\mathbb{R}})$

1: **procedure** MHE_FIT $$({\mathcal{M}},X,y)$$

2: $\widetilde{X}\leftarrow (X\parallel {\bf{1}})$ ⊳Append bias column locally at each party

3: $[C]\leftarrow {\widetilde{X}}^{\top }\times \widetilde{X}$ ⊳Additively shared local partitions ${\widetilde{X}}^{\top }\times \widetilde{X}$

4: $[R]\leftarrow {\widetilde{X}}^{\top }\times y$ ⊳Additively shared local partitions of ${\widetilde{X}}^{\top }\times y$

5: **if** ${\mathtt{len}}({\widetilde{X}}^{\top }) < 4$ **then** ⊳Closed-form solution

6: **R** ← aggregate([*R*])

7: $$[{{\bf{w}}}_{{\mathcal{M}}}]\,\,\,\,\leftarrow {([C])}^{-1}\times {\bf{R}}$$

8: **else** ⊳Batched gradient descent

9: **for** $$j=\overline{0,\ldots {e}_{{\mathcal{M}}}}$$ **do**

10: $${{\bf{w}}}_{{\mathcal{M}}}\leftarrow {\mathtt{aggregate}}([{{\bf{w}}}_{{\mathcal{M}}}])$$

11: $$[{{\bf{w}}}_{{\mathcal{M}}}]\leftarrow [{{\bf{w}}}_{{\mathcal{M}}}]+([R]-[C]\times {{\bf{w}}}_{{\mathcal{M}}})\cdot {\eta }_{{\mathcal{M}}}$$

12: **end** **for**

13: **end** **if**

14: **end** **procedure**

#### Algorithm 8

Logistic regression via MHE (using batched instead of mini-batched gradient descent to simplify)

INPUT:

*N*: number of CKKS slots

${\mathcal{C}}$: CKKS ciphertexts space (i.e., ${({{\mathbb{Z}}}_{p}[X]/(X+1))}^{2}$)

$X\in {{\mathbb{R}}}^{{m}_{i}\times n}$: training data partition held locally at *i*th party

$y\in {{\mathbb{R}}}^{{m}_{i}\times 1}$: training labels partition held locally at *i*th

${\mathcal{M}}$: logistic regression model that stores initial, aggregated weights $\left([{{\bf{w}}}_{{\mathcal{M}}}]\right.\in {{\mathcal{C}}}^{\lceil (n+1)/N\rceil \times 1}$, number of training epochs $({e}_{{\mathcal{M}}}\in {\mathbb{N}})$, and step size $({\eta }_{{\mathcal{M}}}\in {\mathbb{R}})$

1: **procedure** SMC_FIT $$({\mathcal{M}},X,y)$$

2: $\widetilde{X}\leftarrow (X\parallel {\bf{1}})$ ⊳Append bias column locally at each party

3: $$\widetilde{{\bf{X}}}\leftarrow {\mathtt{aggregate}}(\widetilde{X})$$

4: $${\widetilde{{\bf{X}}}}^{\top }\leftarrow {\mathtt{aggregate}}({\widetilde{X}}^{\top })$$

5: **for** $$j=\overline{0,\ldots {e}_{{\mathcal{M}}}}$$ **do**

6: $$[{\bf{P}}]\leftarrow \widetilde{{\bf{X}}}\times [{{\bf{w}}}_{{\mathcal{M}}}]$$

7: [**A**] ← *σ*_*cheby*_([**P**], (0, 1))

8: $$[{{\bf{w}}}_{{\mathcal{M}}}]\leftarrow [{{\bf{w}}}_{{\mathcal{M}}}]-{\widetilde{{\bf{X}}}}^{\top }\times ([{\bf{A}}]-y)\cdot {\eta }_{{\mathcal{M}}}$$

9: **end** **for**

10 **end** **procedure**

We implemented both SMC- and MHE-MICE in Sequre^24,25^—a Codon-based^37^, Pythonic domain-specific language for high-performance SMC computing—in less than 550 lines of high-level Pythonic code. To enable compiling to MHE, we extended Sequre with support for specialized distributed data types and compiler optimization passes to orchestrate multiparty HE computing and automatically handle workload distribution, data aggregation, and other intrinsic properties of HE, such as ciphertext maintenance and encoding^29^. Specifically, our compile-time optimization passes reduce the multiplication depth of the arithmetic expressions, prioritize computing on non-encrypted over the more expensive, encrypted data, and find an optimal aggregation and encoding strategy for the distributed data types. The distributed data types enable arithmetic on top of the private data collectively stored at multiple computing parties, where the data is kept in a non-encrypted form at each party and only partially encrypted when needed throughout the computation. Finally, to enable the essential homomorphic encryption operations (encryption, addition, multiplication, and rotation) and distributed HE operations such as collective bootstrapping, decryption and switching to secret sharing, we re-implemented Lattigo’s^38^ distributed CKKS scheme in Codon.

## Data Availability

The data in the simulation studies can be generated through our data-generating scripts at https://github.com/0xTCG/secure-mice, by running the applications/offline/mi.ipynb notebook. The Georgia Coverdell Acute Stroke Registry (GCASR) data is available by request only and requires approval from GCASR. The Medical Information Mart for Intensive Care (MIMIC) dataset is publicly available.
